# SARS-CoV-2 infection among healthcare workers in South Africa: a longitudinal cohort study

**DOI:** 10.1093/cid/ciab398

**Published:** 2021-05-05

**Authors:** Marta C Nunes, Vicky L Baillie, Gaurav Kwatra, Sutika Bhikha, Charl Verwey, Colin Menezes, Clare L Cutland, David P Moore, Ziyaad Dangor, Yasmin Adam, Rudo Mathivha, Sithembiso C Velaphi, Merika Tsitsi, Ricardo Aguas, Shabir A Madhi, Bara HCW Study Group

**Affiliations:** 1 South African Medical Research Council, Vaccines and Infectious Diseases Analytics Research Unit, School of Pathology, Faculty of Health Sciences, University of the Witwatersrand, Johannesburg, South Africa; 2 Department of Science and Technology/National Research Foundation, South African Research Chair Initiative in Vaccine Preventable Diseases, Faculty of Health Sciences, University of the Witwatersrand, Johannesburg, South Africa; 3 Department of Paediatrics and Child Health, Chris Hani Baragwanath Academic Hospital, Faculty of Health Sciences, University of the Witwatersrand, Johannesburg, South Africa; 4 Department of Internal Medicine, Chris Hani Baragwanath Academic Hospital, Faculty of Health Sciences, University of the Witwatersrand, Johannesburg, South Africa; 5 Department of Obstetrics & Gynaecology, Chris Hani Baragwanath Academic Hospital, Faculty of Health Sciences, University of the Witwatersrand, Johannesburg, South Africa; 6 Department of Intensive Care, Chris Hani Baragwanath Academic Hospital, Faculty of Health Sciences, University of the Witwatersrand, Johannesburg, South Africa; 7 Centre for Tropical Medicine and Global Health, Nuffield Department of Medicine, University of Oxford, Oxford, United Kingdom; 8 African Leadership in Vaccinology Expertise, Faculty of Health Sciences, University of the Witwatersrand, Johannesburg, South Africa

**Keywords:** COVID-19, SARS-CoV-2, hospital staff, healthcare workers, Africa

## Abstract

From April to September 2020, we investigated SARS-CoV-2 infections in a cohort of 396 healthcare workers (HCWs) from five departments at Chris Hani Baragwanath Hospital, South Africa. Overall, 34.6% of HCWs had PCR-confirmed SARS-CoV-2 infection (132.1 [95%CI: 111.8, 156.2] per 1,000 person-months), an additional 27 infections were identified by serology. HCWs in the Internal Medicine department had the highest rate of infection (61.7%). Among PCR-confirmed cases, 10.4% remained asymptomatic, 30.4% were pre-symptomatic and 59.3% symptomatic.

## Introduction

Healthcare workers (HCWs) are in the frontline of the coronavirus disease 2019 (COVID-19) outbreak response and consequently are at higher risk of infection by Severe Acute Respiratory Syndrome Coronavirus 2 (SARS-CoV-2) than the general population[1]. Understanding HCWs' SARS-CoV-2 exposure and risk of infection is critical for characterizing virus transmission patterns, risk-factors for infection, and to inform the effectiveness of infection prevention and control practices. A modelling exercise from the United Kingdom suggested that weekly screening of HCWs for SARS-CoV-2 infection irrespective of symptomatology, could reduce transmission by 16%-23%, if results were available within 24-hours[2]. Studies from Europe reported rates of SARS-CoV-2 polymerase chain reaction (PCR) positivity in up to 24% of symptomatic and 7.1% asymptomatic HCWs[3-5]. A cross-sectional study from Egypt among asymptomatic HCWs identified a 14.3 % PCR positivity rate at the height of the pandemic in the country[6]. Paired serologic testing for SARS-CoV-2 antibodies, can supplement PCR testing. Cross-sectional seroprevalence surveys among HCWs in Europe and the USA during the first wave of the COVID-19 outbreak, reported sero-positivity prevalence between 4% to 24%[7-10]. Similarly, a study in Cape Town, South Africa, reported 10.4% seroprevalence among HCWs from paediatric facilities enrolled between May and July 2020[11].

This longitudinal cohort surveillance of HCWs aimed to determine the incidence of SARS-CoV-2 infection and describe the clinical presentation thereof among HCWs at a large tertiary care hospital in South Africa, during the first COVID-19 wave.

## Methods

### *Study design*

We enrolled HCWs across five departments, including Internal Medicine (IM), Intensive Care, Paediatrics, Obstetrics & Gynaecology, and the Vaccines and Infectious Diseases Analytics (VIDA) research unit, at Chris Hani Baragwanath Academic Hospital (CHBAH) in South Africa which is Africa's largest hospital. Details of the epidemic progression and management of COVID-19 cases at CHBAH are outlined in the supplement. Enrolment occurred between 22nd April to 19th June 2020, whilst VIDA staff were enrolled until 24th July. The current analysis was censored to events occurring until 15th September 2020. Nasal mid-turbinate swabs were collected weekly for PCR testing, irrespective of symptoms suggestive of COVID-19. Venous blood samples were collected at the time of enrolment and every two weeks thereafter. For the current analysis, serology testing was done on the blood samples obtained at enrolment and last study visit. HCWs who tested PCR-positive completed a daily symptoms log for the following 10-days. SARS-CoV-2-infected participants had repeat swabs approximately 2-4 days until at least two consecutive negative tests. Classification of participants accordingly to symptoms in the supplement.

*Laboratory methods*

Details in supplement. Reverse-transcriptase PCR results were classified as positive for SARS-CoV-2 when both the nucleocapsid genes (N1 and N2) were detected at cycle threshold (Ct) value <40. Results were classified as inconclusive if only one of N1 or N2 had Ct values <40.

Serum/plasma samples were tested by an in-house Luminex assay based on reactivity to the receptor binding domain (RBD) of the SARS-CoV-2 spike protein. 45AU/mL was selected as the threshold indicative of SARS-CoV-2 exposure, based on the highest value of RBD IgG in samples collected pre-COVID-19.

*Statistical analysis*

Additional details in supplement. Incidence of PCR-confirmed SARS-CoV-2 infection was calculated using Poisson regression models. The cumulative SARS-CoV-2 infection rate was estimated at the end of follow-up based on a positive PCR result or sero-response. The association between risk of SARS-CoV-2 infection, and participants' characteristics, was estimated by univariate and multivariate generalized linear models. Significance was assessed based on the overlap of the 95% confidence intervals (CIs). Mean PCR Ct values at either diagnosis or lowest recorded and length of infection were compared between participants with different symptomatology, through Student t-tests. P-values ≤0.05 were considered significant.

## *Ethical considerations*

The study was approved by the Human Research Ethics Committee of the University of the Witwatersrand (200405). All study participants provided written informed consent.

## **Results**

Overall, 396 HCWs were enrolled, including 167 (42.2%) from the IM department. The mean age was 38.0 (±9.4) years, 82.6% were female and the majority were black-Africans. Fifty-seven percent reported having at least one comorbidity, including 38.8% being obese (body mass index >30) and 13.2% with hypertension; Table-S1. From 22$^{nd}$ April to 15$^{th}$ September 2020, 137 (34.6%) HCWs were confirmed to be infected by SARS-CoV-2 on PCR testing, for an incidence density of 132.1 (95%CI: 111.8, 156.2) per 1,000 person-months. The epidemic trajectory curve across the five departments overlapped (Figure-S1), with 115 (83.9%) PCR-confirmed cases being detected between 15$^{th}$ June and 19$^{th}$ July 2020. The frequency of PCR-confirmed SARS-CoV-2 infection among HCWs from the IM department was 50.9% (85/167), with an incidence density of 203.0 (95%CI: 164.1, 251.0) cases per 1,000 person-months. Compared to IM, lower incidence was observed for staff from other departments (overall 22% [52/229], 84.1 [95%CI: 64.1, 110.4] cases per 1,000 person-months), and specifically from Paediatrics and Intensive Care; Table-S2.

Of the PCR-confirmed infections, 135 (98.5%) had information regarding symptoms at time of diagnosis, of whom 55 (40.7%) were asymptomatic and 80 (59.3%) had symptoms suggestive of COVID-19. Forty-one (74.6%) of the initially asymptomatic, subsequently developed symptoms within 10-days of SARS-CoV-2 diagnosis (pre-symptomatic), thus 14 (10.4%) were true asymptomatic cases. The frequency of the common symptoms is shown in Table-S3. Symptomatic participants had lower N1 PCR Ct values at diagnosis (mean: 24.2±6.5) and reached lower values (mean: 22.0±4.8) during the course of infection, compared to those who remained asymptomatic (28.9±7.4, p=0.015 and 28.8±7.2, p<0.001, respectively) or pre-symptomatic (28.0±5.5, p=0.002 and 25.1±4.9, p=0.001). Accordingly, in a lower percentage of symptomatic participants (7.6%), SARS-CoV-2 was persistently detected at Ct values ≥30 than asymptomatic (42.9%, p<0.001); Table-S4.

Overall, SARS-CoV-2 was detected for a mean of 17.9 (±8.8) days in nasal swabs. Detection was longer in symptomatic participants at the time of diagnosis (18.9±8.5 days), than those remaining asymptomatic (13.0±6.0 days, p=0.040); Table-S4 and Figure-S2.

Blood samples for serology testing were available from 395 (99.7%) HCWs at the time of enrolment, and four were sero-positive but SARS-CoV-2 PCR-negative. Despite being sero-positive at enrolment, two (50%) subsequently had SARS-CoV-2 identified by PCR 24- and 69-days post-enrolment. There were 135 PCR-confirmed SARS-CoV-2 cases evaluable for sero-response, and all but three (2.2%) demonstrated sero-response >30 days after diagnosis. Additionally, 27 (12.8%) of the 211 HCWs who did not have a positive PCR result and had serology testing at the end of the follow-up demonstrated sero-response. The concordance between PCR-positivity and sero-response was 91.3% (kappa=0.82).

Overall, 166 (41.9%) HCWs had evidence of SARS-CoV-2 infection including 103 (61.7%) from IM and 63 (27.5%) working in other departments. In univariate analysis, working in the IM department, being a nurse, black-African, female, older than 38 years, being hypertensive or obese and using public transport to work were associated with increased risk of SARS-CoV-2 infection. Conversely, receipt of influenza vaccine and smoking were associated with decreased risk. In multivariate analysis, however, only working in the IM department was associated with increased risk, with participants from other departments having an adjusted odds ratio of 0.29 (95%CI: 0.17, 0.49); Table.

**Discussion**

In this longitudinal study, we evaluated the risk of SARS-CoV-2 infection at a large academic hospital in South Africa from end-April through mid-September 2020. The rate of PCR-confirmed SARS-CoV-2 infection across the study population was 34.6%, which increased to 41.9% when including HCW sero-positive at enrolment or sero-response during the study.

Although multiple studies have investigated SARS-CoV-2 infection among HCWs and suggested that exposure to COVID-19 patients poses increased risk, the rates identified in our study are higher than those previously described in Europe and the USA using either PCR testing or serology surveys (4% to 24%)[3, 4, 7-10]. At CHBAH, new strategies were developed to accommodate the unexpected increase in number of patients, including establishing specific wards for suspected and confirmed COVID-19 cases. The majority, of these wards, however, had poor ventilation and lacked isolation cubicles for individual patient management. These characteristics, together with crowded wards, likely contributed to these HCWs being at greater risk of SARS-CoV-2 infection compared to that reported from high-income countries where hospitals are better resourced with facilities to reduce the risk of infections. Moreover, owing to the global shortage of high-quality filtering facepiece respirators, alternate products were sourced. A preliminary study contemporaneous with ours evaluating 12 brands of KN95 masks available in South Africa found that none of the brands met stipulated safety requirements, including mask material filtration efficacy and passing a seal and a qualitative fitting test[12].

We identified a differential risk of SARS-CoV-2 infection, with HCWs from the IM wards having the highest rate of infection. This group was the one mostly exposed to COVID-19 patients; other reasons for the differential rate of infection might be accessibility to training, space and type of personal protective equipment usage. Although in univariate analysis we observed varying risk associated with job category and demographic factors, only working in the IM department remained a significant risk-factor in multivariate analysis.

We identified a 97.8% sero-response rate using the RBD IgG assay among HCWs with PCR-confirmed SARS-CoV-2 infection, which is consistent with previous reports[10]. When the respiratory samples of the three participants who failed to mount an immune response were re-tested, all of them were positive for N1 and N2, and two participants had PCR-positive samples collected at different time-points, confirming true PCR-positivity. The lack of antibodies against RBD does not imply lack of protection from future infections, since they might have produced antibodies against other targets and be protected by other components of the immune system. We identified two PCR-confirmed SARS-CoV-2 infections in participants with detectable antibodies against RBD prior to diagnosis. These re-infections were detected in early June and July before a new SARS-CoV-2 variant was identified in South Africa[13]. The antibody threshold defined during our Luminex assay validation was based on the identification of specific RBD IgG in clinical specimens and it does not necessarily corresponds to a correlate of protection required for functional immunity, and higher levels of antibodies may be required to prevent upper respiratory tract re-infections.

In our study, 40.1% of participants were asymptomatic at the time of diagnosis, and 10.4% did not develop any symptoms within 10-days of diagnosis. Asymptomatic SARS-CoV-2 infections had lower viral load and shorter shedding duration, compared to symptomatic infections. A limitation of our study, however, is that detection of viral RNA does not necessarily imply presence of infectious viruses in the respiratory tract. Nevertheless, current data suggest that viable virus is not shed beyond 20 days after symptom onset, with the probability of detecting live virus significantly decreasing after 5 days[14, 15]. Other study limitations are discussed in the supplement.

Providing HCWs with data about their SARS-CoV-2 virus exposure is important, so that they can protect themselves, their patients, colleagues, and families; protecting HCWs is of paramount importance to fight this epidemic. Longitudinal studies like ours are important to understand the crucial correlation of SARS-CoV-2 antibody levels and protection against re-infection and possibly implications for immunity against new viral variants. In a relatively well-resourced setting in Africa, we identified a very high force of infection suggesting that is likely to be even higher elsewhere in the continent.

NOTES:

Acknowledgements: The authors would like to express special appreciation to the study participants. We also thank Florian Krammer at Icahn School of Medicine at Mount Sinai, New York, USA, for providing the RBD plasmid and Penny Moore's laboratory at the National Institute for Communicable Diseases, South Africa for the expression and purification of the RBD protein. The research reagents for SARS-CoV-2 RNA (NIBSC 20/130) and COVID-19 convalescent plasma panel (NIBSC 20/118) were obtained from the National Institute for Biological Standards and Control, UK. Also special thanks to all the VIDA staff.

Bara HCW Study Group: Firdose Nakwa[3], Sarah Van Blydenstein[4], Michelle Venter[4], Denasha Reddy[4], Jeanine du Plessis[1], Matt Laubscher[1], Lara van der Merwe[1], Nkululeko Mbele[1], Beya Mukendi[1], Shakeel McKenzie[1], Sihle Mtshali[1], Christian Kabasele Mukendi[1], Ayanda Nzimande[1], Wendy Zimkhitha Mandindi[1], Amit Jawaharlal Nana[1], Martin Mosotho Rafuma[1], Masego Nicole Mathibe[1], Andrew Moultrie[1]

Funding statement: This study was supported by the European & Developing Countries Clinical Trials Partnership (grant number RIA2020EF-3020) and The Bill & Melinda Gates Foundation (grant number INV018148_2020). There was also partial support from the Department of Science and Technology and National Research Foundation: South African Research Chair Initiative in Vaccine Preventable Diseases; and the South African Medical Research Council.

Conflict of interest statement: MCN has grants from the European & Developing Countries Clinical trials partnership, Bill & Melinda Gates Foundation, and Pfizer; and has received personal fees from Pfizer and Sanofi Pasteur for expert advice. CLC has grants from the Bill & Melinda Gates Foundation, and has received personal fees from Pfizer, SAM has grants from Bill & Melinda Gates Foundation, Pfizer, Minervax and GSK, and has received personal fees from Bill & Melinda Gates Foundation. All other authors declare no conflict of interest.

| | SARS-CoV-2 infection N=166 | No SARS-CoV-2 infection N=230 | Odds Ratio (95%CI) | Adjust Odds Ratio (95%CI) |
|---|---|---|---|---|
| **Department** | | | | |
| Internal Medicine | 103 (62.1) | 64 (27.8) | reference | reference |
| Pediatrics | 31 (18.7) | 62 (27.0) | 0.31 (0.18, 0.53) | |
| Intensive care | 11 (6.6) | 38 (16.5) | 0.18 (0.09, 0.38) | |
| Obstetrics | 3 (1.8) | 20 (8.7) | 0.09 (0.03, 0.33) | |
| VIDA | 18 (10.8) | 46 (20.0) | 0.24 (0.13, 0.46) | |
| Other than Internal Medicine | 63 (38.0) | 166 (72.2) | 0.24 (0.15, 0.36) | 0.29 (0.17, 0.48) |
| **Job category** | | | | |
| Nurse | 111 (66.9) | 82 (35.7) | reference | reference |
| Physician | 36 (21.7) | 96 (41.7) | 0.28 (0.17, 0.45) | |
| Para-medical | 1 (0.6) | 6 (2.6) | 0.12 (0.01, 1.04) | |
| VIDA Clinical staff | 16 (9.6) | 26 (11.3) | 0.45 (0.23, 0.90) | |
| VIDA Laboratory staff | 2 (1.2) | 20 (8.7) | 0.07 (0.02, 0.32) | |
| Other than nurse | 55 (33.1) | 148 (64.4) | 0.27 (0.18, 0.42) | 0.72 (0.37, 1.42) |
| **Race** | | | | |
| Black-African | 139 (83.7) | 140 (60.9) | reference | reference |
| Asian | 16 (9.6) | 41 (17.8) | 0.39 (0.21, 0.73) | |
| White | 10 (6.0) | 39 (17.0) | 0.26 (0.12, 0.54) | |
| Other | 1 (0.6) | 10 (4.4) | 0.10 (0.01, 0.80) | |

| | | | | |
|---|---|---|---|---|
| Other than Black-African | 27 (16.3) | 90 (39.1) | 0.30 (0.19, 0.49) | 0.62 (0.30, 1.26) |
| **Gender** | | | | |
| Female | 148 (89.2) | 179 (7.7) | reference | reference |
| Male | 18 (10.8) | 51 (22.2) | 0.43 (0.24, 0.76) | 0.87 (0.44, 1.72) |
| **Age** | | | | |
| <38 years old | 71 (42.8) | 143 (62.2) | reference | reference |
| ≥38 years old | 95 (57.2) | 87 (37.8) | 2.20 (1.46, 3.30) | 1.25 (0.75, 2.10) |
| **Transport to work** | | | | |
| Public transport | 78 (47.0) | 61 (26.5) | reference | reference |
| Private car | 84 (50.6) | 166 (72.2) | 0.40 (0.26, 0.61) | |
| Other | 4 (2.4) | 3 (1.3) | 1.04 (0.22, 4.83) | |
| Other than public transport | 88 (53.0) | 169 (73.5) | 0.41 (0.27, 0.62) | 0.80 (0.48, 1.35) |
| **Smoking**[a] | | | | |
| No | 159 (95.8) | 197 (85.7) | reference | reference |
| Yes | 7 (4.2) | 33 (14.3) | 0.26 (0.11, 0.61) | 0.47 (0.17, 1.19) |
| **Influenza vaccination in the current season** | | | | |
| No | 96 (57.8) | 96 (41.7) | reference | reference |
| Yes | 70 (42.2) | 134 (58.3) | 0.52 (0.35, 0.78) | 1.24 (0.73, 2.08) |
| **At least 1 comorbidity**[b] | | | | |
| No | 63 (38.0) | 106 (46.3) | reference | |
| Yes | 103 (62.1) | 123 (53.7) | 1.41 (0.94, 2.12) | |
| Body mass index >30 | 75 (45.7) | 77 (33.8) | 1.65 (1.09, 2.49) | 0.97 (0.59, 1.59) |
| Hypertension | 29 (17.5) | 23 (10.0) | 1.90 (1.05, 3.41) | 1.04 (0.52, 2.06) |
| Asthma | 10 (6.0) | 20 (8.7) | 0.67 (0.30, 1.47) | |

| | | | | |
|---|---|---|---|---|
| HIV | 11 (6.6) | 10 (4.4) | 1.55 (0.64, 3.75) | |
| Diabetes | 5 (3.0) | 4 (1.8) | 1.75 (0.46, 6.61) | |
| Sinusitis or allergy | 1 (0.6) | 4 (1.8) | 0.34 (0.04, 3.08) | |
| Tuberculosis | 0 | 4 (1.8) | N/S | |
| Cardiac | 0 | 3 (1.3) | N/S | |
| Pregnancy | 0 | 2 (0.9) | N/S | |
| Other | 2 (1.1) | 8 (3.7) | 0.34 (0.07, 1.61) | |

N/S: Not significant.

[a]Active or previous smoker.

[b]For individual comorbidities the reference is absence of that particular comorbidity.
