# Severe Acute Respiratory Syndrome Coronavirus 2 Infection Among Healthcare Workers in South Africa: A Longitudinal Cohort Study

**DOI:** 10.1093/cid/ciab398

**Published:** 2021-05-05

**Authors:** Marta C Nunes, Vicky L Baillie, Gaurav Kwatra, Sutika Bhikha, Charl Verwey, Colin Menezes, Clare L Cutland, David P Moore, Ziyaad Dangor, Yasmin Adam, Rudo Mathivha, Sithembiso C Velaphi, Merika Tsitsi, Ricardo Aguas, Shabir A Madhi, Bara HCW Study Group, Firdose Nakwa, Sarah Van Blydenstein, Michelle Venter, Denasha Reddy, Jeanine du Plessis, Matt Laubscher, Lara van der Merwe, Nkululeko Mbele, Beya Mukendi, Shakeel McKenzie, Sihle Mtshali, Christian Kabasele Mukendi, Ayanda Nzimande, Wendy Zimkhitha Mandindi, Amit Jawaharlal Nana, Martin Mosotho Rafuma, Masego Nicole Mathibe, Andrew Moultrie

**Affiliations:** 1 South African Medical Research Council, Vaccines and Infectious Diseases Analytics Research Unit, School of Pathology, Faculty of Health Sciences, University of the Witwatersrand, Johannesburg, South Africa; 2 Department of Science and Technology/National Research Foundation, South African Research Chair Initiative in Vaccine Preventable Diseases, Faculty of Health Sciences, University of the Witwatersrand, Johannesburg, South Africa; 3 Department of Paediatrics and Child Health, Chris Hani Baragwanath Academic Hospital, Faculty of Health Sciences, University of the Witwatersrand, Johannesburg, South Africa; 4 Department of Internal Medicine, Chris Hani Baragwanath Academic Hospital, Faculty of Health Sciences, University of the Witwatersrand, Johannesburg, South Africa; 5 Department of Obstetrics & Gynaecology, Chris Hani Baragwanath Academic Hospital, Faculty of Health Sciences, University of the Witwatersrand, Johannesburg, South Africa; 6 Department of Intensive Care, Chris Hani Baragwanath Academic Hospital, Faculty of Health Sciences, University of the Witwatersrand, Johannesburg, South Africa; 7 Centre for Tropical Medicine and Global Health, Nuffield Department of Medicine, University of Oxford, Oxford, United Kingdom; 8 African Leadership in Vaccinology Expertise, Faculty of Health Sciences, University of the Witwatersrand, Johannesburg, South Africa

**Keywords:** COVID-19, SARS-CoV-2, hospital staff, healthcare workers, Africa

## Abstract

From April to September 2020, we investigated severe acute respiratory syndrome coronavirus 2 (SARS-CoV-2) infections in a cohort of 396 healthcare workers (HCWs) from 5 departments at Chris Hani Baragwanath Hospital, South Africa. Overall, 34.6% of HCWs had polymerase chain reaction–confirmed SARS-CoV-2 infection (132.1 [95% confidence interval, 111.8–156.2] infections per 1000 person-months); an additional 27 infections were identified by serology. HCWs in the internal medicine department had the highest rate of infection (61.7%). Among polymerase chain reaction–confirmed cases, 10.4% remained asymptomatic, 30.4% were presymptomatic, and 59.3% were symptomatic.

Healthcare workers (HCWs) are in the frontline of the coronavirus disease 2019 (COVID-19) outbreak response and consequently are at higher risk of infection by severe acute respiratory syndrome coronavirus 2 (SARS-CoV-2) than the general population [1]. Understanding HCWs’ SARS-CoV-2 exposure and risk of infection is critical for characterizing virus transmission patterns, risk factors for infection, and to inform the effectiveness of infection prevention and control practices. A modeling exercise from the United Kingdom suggested that weekly screening of HCWs for SARS-CoV-2 infection, irrespective of symptoms, could reduce transmission by 16%–23%, if results were available within 24 hours [2]. Studies from Europe reported rates of SARS-CoV-2 polymerase chain reaction (PCR) positivity in up to 24% of symptomatic and 7.1% of asymptomatic HCWs [3–5]. 

A cross-sectional study from Egypt among asymptomatic HCWs identified a 14.3% PCR positivity rate at the height of the pandemic in the country [6]. Paired serologic testing for SARS-CoV-2 antibodies, can supplement PCR testing. Cross-sectional seroprevalence surveys among HCWs in Europe and the United States during the first wave of the COVID-19 outbreak reported seropositivity prevalence between 4% and 24% [7–10]. Similarly, a study in Cape Town, South Africa, reported 10.4% seroprevalence among HCWs from pediatric facilities enrolled between May and July 2020 [11]. In the current study, longitudinal cohort surveillance of HCWs aimed to determine the incidence of SARS-CoV-2 infection and describe the clinical presentation thereof among HCWs at a large tertiary care hospital in South Africa, during the first COVID-19 wave.

## METHODS

### Study Design

We enrolled HCWs across 5 departments, including internal medicine (IM), intensive care, pediatrics, obstetrics and gynecology, and the Vaccines and Infectious Diseases Analytics (VIDA) research unit, at Chris Hani Baragwanath Academic Hospital (CHBAH) in South Africa, Africa’s largest hospital. Details of the epidemic progression and management of COVID-19 cases at CHBAH are outlined in the Supplementary Materials. Enrollment occurred between 22 April and 19 June 2020, and the VIDA staff were enrolled until 24 July. The current analysis was censored to events occurring until 15 September 2020. Nasal midturbinate swab samples were collected weekly for PCR testing, irrespective of symptoms suggestive of COVD-19. Venous blood samples were collected at the time of enrollment and every 2 weeks thereafter. For the current analysis, serology testing was done on the blood samples obtained at enrollment and last study visit. HCWs who tested PCR positive completed a daily symptom log for the following 10 days. SARS-CoV-2–infected participants had repeated swab samples obtained approximately every 2–4 days until ≥2 consecutive negative tests. Participants were classified accordingly to symptoms, as detailed in the Supplementary Materials.

### Laboratory Methods

Details are provided in the Supplementary Materials. Reverse-transcriptase PCR results were classified as positive for SARS-CoV-2 when both the nucleocapsid genes (N1 and N2) were detected at a cycle threshold (Ct) value <40. Results were classified as inconclusive if the Ct value was <40 for N1 or N2, but not both. Serum and plasma samples were tested using an in-house Luminex assay based on reactivity to the receptor-binding domain (RBD) of the SARS-CoV-2 spike protein. A value of 45 AU/mL was selected as the threshold indicative of SARS-CoV-2 exposure, based on the highest value of RBD immunoglobulin (Ig) G in samples collected before COVID-19.

### Statistical Analysis

Additional details are provided in the Supplementary Materials. The incidence of PCR-confirmed SARS-CoV-2 infection was calculated using Poisson regression models. The cumulative SARS-CoV-2 infection rate was estimated at the end of follow-up based on a positive PCR result or seroresponse. The association between risk of SARS-CoV-2 infection and participants’ characteristics was estimated by means of univariate and multivariate generalized linear models. Significance was assessed based on the overlap of the 95% confidence intervals (CIs). Mean PCR Ct values at either diagnosis or lowest recorded value and lengths of infection were compared between participants with different symptoms, using Student *t* tests. Differences were considered significant at *P* ≤ .05.

### Ethical Considerations

The study was approved by the Human Research Ethics Committee of the University of the Witwatersrand (no. 200405). All study participants provided written informed consent.

## RESULTS

Overall, 396 HCWs were enrolled, including 167 (42.2%) from the IM department. The mean age (standard deviation [SD]) was 38.0 (9.4) years, 82.6% were female, and the majority were black Africans. Fifty-seven percent reported having ≥1 comorbid conditions, including 38.8% who were obese (body mass index >30 [calculated as weight in kilograms divided by height in meters squared]) and 13.2% with hypertension; Supplementary Table 1. From 22 April to 15 September 2020, 137 (34.6%) HCWs were confirmed with PCR to be infected by SARS-CoV-2, for an incidence density of 132.1 cases (95% CI, 111.8–156.2) per 1000 person-months. The epidemic trajectory curve across the 5 departments overlapped (Supplementary Figure 1), with 115 (83.9%) PCR-confirmed cases detected between 15 June and 19 July 2020. The frequency of PCR-confirmed SARS-CoV-2 infection among HCWs from the IM department was 50.9% (85 of 167), with an incidence density of 203.0 cases (95% CI, 164.1–251.0) per 1000 person-months. Compared with IM, lower incidences were observed for staff from other departments (overall incidence, 22% [52 of 229]; 84.1 cases [95% CI, 64.1–110.4] per 1000 person-months), specifically from pediatrics and intensive care (Supplementary Table 2).

Of the HWCs with PCR-confirmed infections, 135 (98.5%) had information regarding symptoms at time of diagnosis, of whom 55 (40.7%) were asymptomatic and 80 (59.3%) had symptoms suggestive of COVID-19. Forty-one (74.6%) of the initially asymptomatic subsequently developed symptoms within 10 days after SARS-CoV-2 diagnosis (presymptomatic); thus, 14 (10.4%) were true asymptomatic cases. The frequency of the common symptoms is shown in Supplementary Table 3. Symptomatic participants had lower N1 PCR Ct values at diagnosis (mean [SD], 24.2 [6.5]) and reached lower values (22.0 [4.8]) during the course of infection, compared with those who remained asymptomatic (28.9 [7.4] [*P* = .01] and 28.8 [7.2] [*P* < .001], respectively) or presymptomatic (28.0 [5.5] [*P* = .002] and 25.1 [4.9] [*P* = .001]). Accordingly, SARS-CoV-2 was persistently detected at Ct values ≥30 in a lower percentage of symptomatic (7.6%) than asymptomatic (42.9%) participants (*P* < .001) (Supplementary Table 4).

Overall, SARS-CoV-2 was detected for a mean (SD) of 17.9 (8.8) days in nasal swab samples. Detection continued longer in symptomatic participants at the time of diagnosis (mean [SD] 18.9 [8.5] days) than in those remaining asymptomatic (13.0 [6.0] days; *P* = .04) (Supplementary Table 4 and Supplementary Figure 2).

Blood samples for serology testing were available from 395 (99.7%) HCWs at the time of enrollment, and 4 were seropositive but PCR negative for SARS-CoV-2. Despite being seropositive at enrollment, 2 (50%) subsequently had SARS-CoV-2 identified by PCR 24 and 69 days after enrollment. There were 135 PCR-confirmed SARS-CoV-2 cases evaluable for seroresponse, and all but 3 (2.2%) demonstrated seroresponse >30 days after diagnosis. In addition, 27 (12.8%) of the 211 HCWs who did not have a positive PCR result and underwent serology testing at the end of the follow-up demonstrated a seroresponse. The concordance between PCR positivity and seroresponse was 91.3% (κ = 0.82).

Overall, 166 (41.9%) HCWs had evidence of SARS-CoV-2 infection, including 103 (61.7%) from IM and 63 (27.5%) working in other departments. In univariate analysis, working in the IM department, being a nurse, black African, or female, age >38 years, being hypertensive or obese, and taking public transport to work were associated with increased risk of SARS-CoV-2 infection. Conversely, receipt of influenza vaccine and smoking were associated with decreased risk. In multivariate analysis, however, only working in the IM department was associated with increased risk, with participants from other departments having an adjusted odds ratio of 0.29 (95% CI, .17–.49); Table 1.

**Table 1.** Characteristics of Healthcare Workers Enrolled in the Study and Risk of Developing Severe Acute Respiratory Syndrome Coronavirus 2 Infection Detected With Polymerase Chain Reaction or Serology

Characteristic	HCWs, No. (%)		OR (95% CI)	
	SARS-CoV-2 (n = 166)	No SARS-CoV-2 (n = 230)	Unadjusted	Adjusted
Department				
IM	103 (62.1)	64 (27.8)	Reference	Reference
Pediatrics	31 (18.7)	62 (27.0)	0.31 (.18–.53)	…
Intensive care	11 (6.6)	38 (16.5)	0.18 (.09–.38)	…
Obstetrics	3 (1.8)	20 (8.7)	0.09 (.03–.33)	…
VIDA	18 (10.8)	46 (20.0)	0.24 (.13–.46)	…
Other than IM	63 (38.0)	166 (72.2)	0.24 (.15–.36)	0.29 (.17–.48)
Job category				
Nurse	111 (66.9)	82 (35.7)	Reference	Reference
Physician	36 (21.7)	96 (41.7)	0.28 (.17–.45)	…
Paramedical	1 (0.6)	6 (2.6)	0.12 (.01–1.04)	…
VIDA clinical staff	16 (9.6)	26 (11.3)	0.45 (.23–.90)	…
VIDA laboratory staff	2 (1.2)	20 (8.7)	0.07 (.02–.32)	…
Other than nurse	55 (33.1)	148 (64.4)	0.27 (.18–.42)	0.72 (.37–1.42)
Race				
Black African	139 (83.7)	140 (60.9)	Reference	Reference
Asian	16 (9.6)	41 (17.8)	0.39 (.21–.73)	…
White	10 (6.0)	39 (17.0)	0.26 (.12–.54)	…
Other	1 (0.6)	10 (4.4)	0.10 (.01–.80)	…
Other than black African	27 (16.3)	90 (39.1)	0.30 (.19–.49)	0.62 (.30–1.26)
Sex				
Female	148 (89.2)	179 (7.7)	Reference	Reference
Male	18 (10.8)	51 (22.2)	0.43 (.24–.76)	0.87 (.44–1.72)
Age				
<38 y	71 (42.8)	143 (62.2)	Reference	Reference
≥38 y	95 (57.2)	87 (37.8)	2.20 (1.46–3.30)	1.25 (.75–2.10)
Transport to work				
Public transport	78 (47.0)	61 (26.5)	Reference	Reference
Private car	84 (50.6)	166 (72.2)	0.40 (.26–.61)	…
Other	4 (2.4)	3 (1.3)	1.04 (.22–4.83)	…
Other than public transport	88 (53.0)	169 (73.5)	0.41 (.27–.62)	0.80 (.48–1.35)
Smoking^a^				
No	159 (95.8)	197 (85.7)	Reference	Reference
Yes	7 (4.2)	33 (14.3)	0.26 (.11–.61)	0.47 (.17–1.19)
Influenza vaccination in current season				
No	96 (57.8)	96 (41.7)	Reference	Reference
Yes	70 (42.2)	134 (58.3)	0.52 (.35–.78)	1.24 (.73–2.08)
≥1 Comorbid condition^b^				
No	63 (38.0)	106 (46.3)	Reference	…
Yes	103 (62.1)	123 (53.7)	1.41 (.94–2.12)	…
BMI >30^c^	75 (45.7)	77 (33.8)	1.65 (1.09–2.49)	0.97 (.59–1.59)
Hypertension	29 (17.5)	23 (10.0)	1.90 (1.05–3.41)	1.04 (.52–2.06)
Asthma	10 (6.0)	20 (8.7)	0.67 (.30–1.47)	…
HIV	11 (6.6)	10 (4.4)	1.55 (.64–3.75)	…
Diabetes	5 (3.0)	4 (1.8)	1.75 (.46–6.61)	…
Sinusitis or allergy	1 (0.6)	4 (1.8)	0.34 (.04–3.08)	…
Tuberculosis	0	4 (1.8)	NS	…
Cardiac disease	0	3 (1.3)	NS	…
Pregnancy	0	2 (0.9)	NS	…
Other	2 (1.1)	8 (3.7)	0.34 (.07–1.61)	…

Abbreviations: BMI, body mass index; CI, confidence interval; HCWs, healthcare workers; HIV, human immunodeficiency virus; IM, internal medicine; NS, not significant; OR, odds ratio; SARS-CoV-2, severe acute respiratory syndrome coronavirus 2; VIDA, Vaccines and Infectious Diseases Analytics.

^a^Active or previous smoker.

^b^For individual comorbid conditons, the reference is absence of that particular condition.

^c^BMI calculated as weight in kilograms divided by height in meters squared.

## Discussion

In this longitudinal study, we evaluated the risk of SARS-CoV-2 infection at a large academic hospital in South Africa from the end of April through mid-September 2020. The rate of PCR-confirmed SARS-CoV-2 infection across the study population was 34.6%, which increased to 41.9% when including HCWs who were seropositive at enrollment or had a seroresponse during the study.

Although multiple studies have investigated SARS-CoV-2 infection among HCWs and suggested that exposure to patients with COVID-19 poses increased risk, the rates identified in our study are higher than those previously described in Europe and the United States using either PCR testing or serology surveys (4%–24%) [3, 4, 7–10]. At CHBAH, new strategies were developed to accommodate the unexpected increase in number of patients, including establishing specific wards for suspected and confirmed COVID-19 cases. The majority, of these wards, however, had poor ventilation and lacked isolation cubicles for individual patient management. These characteristics, together with crowded wards, likely contributed to these HCWs being at greater risk of SARS-CoV-2 infection than those in studies reported from high-income countries, where hospitals have better resources to reduce the risk of infections. Moreover, owing to the global shortage of high-quality filtering facepiece respirators, alternate products were sourced. A preliminary study contemporaneous with ours evaluating 12 brands of KN95 masks available in South Africa found that none of the brands met stipulated safety requirements, including mask material filtration efficacy and passing a seal and a qualitative fitting test [12].

We identified a differential risk of SARS-CoV-2 infection, with HCWs from the IM wards having the highest rate of infection. This group was the one mostly exposed to patients with COVID-19; other reasons for the differential rate of infection might be accessibility to training, space, and type of personal protective equipment usage. Although in univariate analysis we observed varying risk associated with job category and demographic factors, in multivariate analysis only working in the IM department remained a significant risk factor.

We identified a 97.8% seroresponse rate using the RBD IgG assay among HCWs with PCR-confirmed SARS-CoV-2 infection, which is consistent with previous reports [10]. When the respiratory samples from the 3 participants who failed to mount an immune response were retested, all of them were positive for N1 and N2, and 2 participants had PCR-positive samples collected at different time points, confirming true PCR positivity. The lack of antibodies against RBD does not imply lack of protection from future infections, since participants might have produced antibodies against other targets and be protected by other components of the immune system. We identified 2 PCR-confirmed SARS-CoV-2 infections in participants with detectable antibodies against RBD before diagnosis. These reinfections were detected in early June and July before a new SARS-CoV-2 variant was identified in South Africa [13]. The antibody threshold defined during our Luminex assay validation was based on the identification of specific RBD IgG in clinical specimens; it does not necessarily correspond to a correlate of protection required for functional immunity, and higher levels of antibodies may be required to prevent upper respiratory tract reinfections.

In our study, 40.1% of participants were asymptomatic at the time of diagnosis, and 10.4% did not experience any symptoms within 10 days of diagnosis. Asymptomatic SARS-CoV-2 infections had lower viral load and shorter shedding duration, compared with symptomatic infections. A limitation of our study, however, is that detection of viral RNA does not necessarily imply the presence of infectious viruses in the respiratory tract. Nevertheless, current data suggest that viable virus is not shed beyond 20 days after symptom onset, with the probability of detecting live virus significantly decreasing after 5 days [14, 15]. Other study limitations are discussed in the Supplementary Materials.

Providing HCWs with data about their SARS-CoV-2 virus exposure is important, so they can protect themselves, their patients, colleagues, and families; protecting HCWs is of paramount importance to fight this epidemic. Longitudinal studies like ours are important to understand the crucial correlation of SARS-CoV-2 antibody levels and protection against reinfection and possibly implications for immunity against new viral variants. In a relatively well-resourced setting in Africa, we identified a very high force of infection, suggesting that it is likely to be even higher elsewhere in the continent.

## Supplementary Data

Supplementary materials are available at *Clinical Infectious Diseases online*. Consisting of data provided by the authors to benefit the reader, the posted materials are not copyedited and are the sole responsibility of the authors, so questions or comments should be addressed to the corresponding author.

ciab398_suppl_Supplementary_Materials

## Notes

***Acknowledgments.*** The authors express special appreciation to the study participants. They also thank Florian Krammer, Icahn School of Medicine at Mount Sinai, New York, New York, for providing the receptor-binding domain (RBD) plasmid and Penny Moore’s laboratory at the National Institute for Communicable Diseases, South Africa, for the expression and purification of the RBD protein. The research reagents for severe acute respiratory syndrome coronavirus 2 RNA (NIBSC 20/130) and coronavirus disease 2019 convalescent plasma panel (NIBSC 20/118) were obtained from the National Institute for Biological Standards and Control, United Kingdom. Special thanks to all the Vaccines and Infectious Diseases Research Unit staff.

***Study group.*** The Bara HCW Study Group included Firdose Nakwa (Department of Paediatrics and Child Health, Chris Hani Baragwanath Academic Hospital [CHBAH], Faculty of Health Sciences, University of the Witwatersrand, Johannesburg, South Africa); Sarah Van Blydenstein, Michelle Venter, and Denasha Reddy (Department of Internal Medicine, CHBAH, Faculty of Health Sciences, University of the Witwatersrand); and Jeanine du Plessis, Matt Laubscher, Lara van der Merwe, Nkululeko Mbele, Beya Mukendi, Shakeel McKenzie, Sihle Mtshali, Christian Kabasele Mukendi, Ayanda Nzimande, Wendy Zimkhitha Mandindi, Amit Jawaharlal Nana, Martin Mosotho Rafuma, Masego Nicole Mathibe, and Andrew Moultrie (South African Medical Research Council, Vaccines and Infectious Diseases Analytics Research Unit, School of Pathology, Faculty of Health Sciences, University of the Witwatersrand).

***Financial support.*** This work was supported by the European & Developing Countries Clinical Trials Partnership (grant RIA2020EF-3020), the Bill & Melinda Gates Foundation (grant INV018148_2020), and from the Department of Science and the Technology and National Research Foundation (South African Research Chair Initiative in Vaccine Preventable Diseases), and the South African Medical Research Council.

***Potential conflicts of interest.*** M. C. N. has received grants from the European & Developing Countries Clinical trials partnership, the Bill & Melinda Gates Foundation, and Pfizer and has received personal fees from Pfizer and Sanofi Pasteur for expert advice. C. L. C. has received grants from the Bill & Melinda Gates Foundation and has received personal fees from Pfizer. S. A. M. has received grants from the Bill & Melinda Gates Foundation, Pfizer, Minervax, and GlaxoSmithKline and has received personal fees from the Bill & Melinda Gates Foundation. All other authors report no potential conflicts. All authors have submitted the ICMJE Form for Disclosure of Potential Conflicts of Interest. Conflicts that the editors consider relevant to the content of the manuscript have been disclosed.
