# Supplementary material for: Severe Acute Respiratory Syndrome Coronavirus 2 Infection Among Healthcare Workers in South Africa: A Longitudinal Cohort Study
Source: Clin Infect Dis. 2021 May 5;73(10):1896–900. doi: 10.1093/cid/ciab398 (PMC8135922; doi:10.1093/cid/ciab398)
Supplement: ciab398_suppl_Supplementary_Materials [file ciab398_suppl_supplementary_materials.docx]

**Supplementary material**

**Methods**

We enrolled healthcare workers (HCWs) working at Chris Hani Baragwanath Academic Hospital (CHBAH) in Soweto, Johannesburg, South Africa, into a longitudinal cohort study. CHBAH, which is Africa’s largest tertiary care hospital with over 3000 beds and 6700 staff, is a public sector hospital serving a low-income, predominantly black-African community, estimated at 1.7 million people. The first SARS-CoV-2 case in South Africa was reported on 5th March 2020 and, subsequently, the first known hospitalized case of COVID-19 at CHBAH was diagnosed on 6th April 2020. The number of COVID-19 cases admitted at CHBAH were low until the end of May 2020, after which the numbers rapidly increased with infections peaking from mid-June to early August (week 26 to 32), Figure-S1. We enrolled HCWs who were likely to have direct contact with COVID-19 patients, and laboratory staff responsible for processing respiratory samples from COVID-19 suspected cases, working across five departments, including Internal Medicine, Intensive Care, Paediatrics, Obstetrics & Gynaecology, and the Vaccines and Infectious Diseases Analytics (VIDA) research unit. Six dedicated multi-bed wards were established at CHBAH for COVID-19 suspected or confirmed cases. At the beginning of the pandemic, a 28 bedded medical acute care unit admission ward was established, as well as an Infectious Disease ward, with 19 beds and state-of-the-art facilities, including a high-efficiency particulate air filter and negative pressure systems. As the numbers of COVID-19 cases progressively increased, four additional wards were established, including a ward for treatment with high flow oxygen with 32 adult and six paediatric beds; a 52 beds ward with separate individual cubicles; and two open-plan wards for COVID-19-confirmed patients with 52 beds each. Apart from the Infectious Disease ward, the other wards were open plan with no special ventilation or filtration system. All staff were trained in donning and doffing and had access to PPE. While the shifts were 12 hours long, depending on the staff category, staff were in full PPE for 4-6 hours with the rest of their time being involved in other clinical activities. Similarly, in general paediatrics, two open plan wards were established: one for suspected COVD-19 cases and a 15-bed ward for confirmed COVID-19 cases, including five high care beds with facilities for mechanical ventilation. Forty-seven beds were re-purposed for suspected/COVID-19 women admitted to the obstetric wards. The wards earmarked for children and pregnant women did not have any special ventilation or filtration system. HCWs assigned to the COVID-19 wards were on fixed rotation in those wards, and not allowed to cross-cover to other wards. Nurses and physicians who triaged patients with suspected SARS-CoV-2 infection, and provided care to COVID-19 cases, were considered to be at greatest risk of SARS-CoV-2 infection. Staff from VIDA consisted of clinical staff responsible for collection of samples from COVID-19 suspected cases, and the laboratory staff involved in SARS-CoV-2 PCR testing.

Demographic, health and behavioural questionnaires, including questions on exposure to known COVID-19 cases, symptoms and medication use, were administered at enrolment and at every subsequent weekly visit. Comorbidities were self-reported, although at the end of follow-up participants were offered human immunodeficiency virus (HIV) testing. SARS-CoV-2 PCR results were made available to participants within 24-hours of sampling. Care of participants who tested SARS-CoV-2 PCR-positive was per hospital protocols and included self-isolation for 10-14 days.

Participants were classified as having symptoms suggestive of COVID-19 if they reported any of the following signs or symptoms in the previous month (at enrolment visit), or since the last study visit: fever/feeling feverish, cough, sore throat, rhinitis, myalgia, shortness of breath, acute gastroenteritis/vomiting/nausea, impaired sense of smell or taste. Although fatigue and headache were included in the symptoms list, they were not used to categorize participants as symptomatic in the absence of any other symptom listed above. Pre-symptomatic participants were defined as those who were asymptomatic at the time of SARS-CoV-2 infection diagnosis, but later developed symptoms in the subsequent 10-days.

*SARS-CoV-2 PCR testing*

Total nucleic acids were extracted from respiratory specimens using an automated NucliSENS-easyMAG nucleic acid extraction platform. Reverse-transcriptase PCR using emergency use authorization developed assays, based on the Centers for Disease Control and Prevention, was used.

*Testing for SARS-CoV-2 antibodies*

Serum or plasma samples were tested by an in-house Luminex assay based on reactivity to the receptor binding domain (RBD) of the immunogenic SARS-CoV-2 spike protein. Expression plasmids encoding SARS-CoV-2 RBD were obtained from Krammer’s laboratory, Mount Sinai, USA. Recombinant RBD protein was expressed as described previously(1) and was coupled to magnetic microsphere beads (Bio-Rad, USA), using a two-step carbodiimide reaction(2). An in-house interim reference serum was developed by pooling convalescent serum from COVID-19 patients. This interim reference was calibrated against research reagent NIBSC 20/130 distributed by the National Institute for Standards and Biological Control (NIBSC, Potters Bar, UK, https://www.nibsc.org/). The interim Arbitrary Units (AU) value assigned to the in-house reference serum was 2442AU/mL for RBD IgG. Adult serum samples collected prior to 2020 (n=37) were used to assess specificity. 45AU/mL was selected as the threshold indicative of SARS-CoV-2, based on the highest value of RBD IgG in samples from pre-COVID-19. Sensitivity of the assay in detecting past or current infection was assessed using serum samples obtained from randomly selected (n=15) participants who tested SARS-CoV-2 PCR-positive in this study and who had serial sampling before and after post-symptom onset, including cases with mild-moderate illness and asymptomatic infections. The sensitivity of the IgG assay was 75% for samples collected 7-14 days and 100 % for samples collected 14 days following the first PCR-positive SARS-CoV-2 test.

The assay was also evaluated against a COVID-19 convalescent plasma panel (NIBSC code 20/118) intended for the development and evaluation of serological assays for the detection of antibodies against SARS-CoV-2. The optimal serum/plasma and secondary antibody dilutions for the assay were 1:100 and 1:200, respectively. The over range samples were re-tested at higher dilutions (1:200-1:1000). Samples were analysed in true duplicate, and each plate included high- and low-control sera. Bead fluorescence was read with the Bio-Plex 200 instrument (Bio-Rad) using Bio-Plex manager5.0 software (Bio-Rad). Serology testing was only done at the end of follow-up, and results were unavailable to the participants.

*Statistical analysis*

Participants’ characteristics were described as percentages or means with standard deviations (SD). Participants were classified as sero-positive at enrolment if RBD IgG >45AU/mL, and sero-responders if at end of follow-up they had RBD IgG >45AU/mL with concomitant ≥4-fold increase from enrolment. Sero-response was evaluated in all participants with PCR-confirmed infection, and in those with a last blood draw after August 1^st^ 2020. Concordance in diagnosing SARS-CoV-2 infection by PCR, or based on sero-response, was assessed by Cohen's Kappa method.

Mean PCR Ct values were used as a proxy of the amount of viral RNA present. The duration of SARS-CoV-2 RNA detection by PCR, as a crude measure of viral shedding, was calculated as the number of days from the time of collection of the first positive swab to the time of the first of two consecutive negative swabs. Only participants with a first negative swab collected within seven days of the previous positive or inconclusive swab were included in the calculation of the length of infection. Since low Ct values suggest shedding of live virus (3, 4) analyses considering the time from diagnosis to Ct values ≥30 and the percentage of participants with Ct values persistently ≥30 were conducted.

**Discussion**

Study limitations include that all demographic and clinical data were self-reported and consequently subject to recall bias, and other artifacts such as the willingness of the participants to disclose comorbidities. For example, while the prevalence of HIV infection in South African adults is approximately 19% (5), only 4% of the study participants reported being infected with HIV. Although we offered HIV testing at the end of the study, many participants declined testing for HIV. We enrolled only a sub-sample of the hospital staff and not all HCWs. In addition, sero-response was assessed against only one domain of the SARS-CoV-2 spike protein; however, our extensive Luminex assay validation showed excellent specificity and sensitivity. Since we used only two time-points to describe sero-response, we might have missed early PCR-negative infections, the antibodies from which might have waned by the end of follow-up, although levels of anti-spike IgG have been described to be sustained for at least six months (6).

**Table-S1.** Characteristics of the healthcare workers enrolled in the study

|  | **Overall**  **N=396** | **Internal Medicine**  **N=167** | **Other departments**  **N=229** |
| --- | --- | --- | --- |
| **Department** |  |  |  |
| Internal Medicine | 167 (42.2) |  |  |
| Pediatrics | 93 (23.5) |  |  |
| Intensive care | 49 (12.4) |  |  |
| Obstetrics | 23 (5.8) |  |  |
| VIDA | 64 (16.2) |  |  |
| **Job category** |  |  |  |
| Nurse | 193 (48.7) | 128 (76.7) | 65 (28.4) |
| Physician | 132 (33.3) | 38 (22.8) | 94 (41.1) |
| Para-medical | 7 (1.8) | 1 (0.6) | 6 (2.6) |
| VIDA Clinical staff | 42 (10.6) | N/A | 42 (18.3) |
| VIDA Laboratory staff | 22 (5.6) | N/A | 22 (9.6) |
| **Race** |  |  |  |
| Black-African | 279 (70.5) | 136 (81.4) | 143 (62.5) |
| Asian | 57 (14.4) | 19 (11.4) | 38 (16.6) |
| White | 49 (12.4) | 8 (4.8) | 41 (17.9) |
| Other | 11 (2.8) | 4 (2.4) | 7 (3.1) |
| **Female** | 327 (82.6) | 146 (87.4) | 181 (79.0) |
| **Mean age in years (SD)** | 38.0 (9.4) | 40.0 (8.7) | 36.6 (9.6) |
| **Transport to work** |  |  |  |
| Private car | 250 (63.1) | 84 (50.3) | 166 (72.5) |
| Public transport | 139 (35.1) | 82 (49.1) | 57 (24.9) |
| Other | 7 (1.8) | 1 (0.6) | 6 (2.6) |
| **At least 1 comorbidity^a^** | 226 (57.2) | 110 (65.9) | 116 (50.9) |
| Body mass index >30 | 152 (38.8) | 79 (47.9) | 73 (32.2) |
| Hypertension | 52 (13.2) | 30 (18.0) | 22 (9.7) |
| Asthma | 30 (7.6) | 11 (6.6) | 19 (8.3) |
| HIV^b^ | 21 (5.3) | 13 (7.8) | 8 (3.5) |
| Diabetes | 9 (2.3) | 5 (3.0) | 4 (1.8) |
| Sinusitis or allergy | 5 (1.3) | 2 (1.2) | 3 (1.3) |
| Tuberculosis | 4 (1.0) | 1 (0.6) | 3 (1.3) |
| Cardiac | 3 (0.8) | 0 | 3 (1.3) |
| Pregnancy | 2 (0.5) | 0 | 2 (0.9) |
| Other | 10 (2.5) | 3 (1.8) | 7 (3.1) |
| **Smoking** |  |  |  |
| Active smoker | 25 (6.3) | 8 (4.8) | 17 (7.4) |
| Previous smoker | 15 (3.8) | 3 (1.8) | 12 (5.2) |
| **Received influenza vaccine** | 204 (51.5) | 48 (28.7) | 156 (68.1) |

Results are n (%) unless stated otherwise.

SD: Standard deviation.

N/A: Not applicable.

^a^Information available for 395 participants.

^b^16 HCWs self-reported to be HIV-infected and an additional 5 had a reactive ELISA test out of 199 tested.

**Table-S2.** Positivity rate and incidence of PCR-confirmed SARS-CoV-2 infection among healthcare workers from the five departments

| **Department** | **Positivity Rate**  **n/N (%)** | **Incidence per 1000 person-months (95%CI)** |
| --- | --- | --- |
| Overall | 137/396 (34.6) | 132.1 (111.8, 156.2) |
| Internal Medicine | 85/167 (50.9) | 203.0 (164.1, 251.0) |
| Other than Internal Medicine | 52/229 (22.7) | 84.1 (64.1, 110.4) |
| Pediatrics | 25/93 (26.9) | 91.3 (61.7, 135.0) |
| Intensive care | 10/49 (20.4) | 62.7 (33.7, 116.5) |
| Obstetrics | 2/23 (8.7) | 43.8 (11.0, 175.2) |
| VIDA | 15/64 (23.4) | 107.0 (65.1, 179.1) |

**Table-S3.** Self-reported symptoms by healthcare workers at the time SARS-CoV-2 PCR detection or within 10 days of detection by those who were diagnosed in the pre-symptomatic phase

| **Symptoms** | **Symptoms at diagnosis**  **N=80**  **n (%)** | **Symptoms developed by pre-symptomatic HCWs in the 10 days following diagnosis**  **N=41**  **n (%)** | **Symptoms at diagnosis or developed within 10 days of diagnosis**  **N=123^a^**  **n (%)** |
| --- | --- | --- | --- |
| Cough | 48 (60.0) | 28 (68.3) | 94 (76.4) |
| Impaired sense of smell or taste | 25 (31.3) | 30 (73.2) | 90 (73.2) |
| Sore throat | 43 (53.8) | 23 (56.1) | 83 (67.5) |
| Myalgia | 21 (26.3) | 29 (70.7) | 81 (65.9) |
| Rhinitis / runny nose | 42 (52.5) | 14 (34.1) | 69 (56.1) |
| Acute gastroenteritis/vomiting/nausea | 7 (8.8) | 20 (48.8) | 54 (43.9) |
| Fever / feeling feverish | 28 (35.0) | 11 (26.8) | 52 (42.3) |
| Shortness of breath | 12 (15.0) | 15 (36.6) | 50 (40.7) |
| Headache^b^ | 48 (60.0) | 10 (24.4) | 65 (52.8) |
| Fatigue^b^ | 35 (43.8) | 30 (73.2) | 90 (73.2) |

^a^Two participants had missing information at the time of PCR detection but had symptoms reported in the dairy card.

^b^Not included to categorize participants as symptomatic.

**Table-S4.** SARS-CoV-2 PCR characteristics among healthcare workers who were symptomatic, asymptomatic or pre-symptomatic at the time of diagnosis

|  | **Symptomatic** | **Asymptomatic** | **Pre-symptomatic** | **p-value^a^** | **p-value^b^** | **p-value^c^** |
| --- | --- | --- | --- | --- | --- | --- |
| **Mean PCR Ct value at diagnosis, (SD)**^d^ | 24.2 (6.5)  [79] | 28.9 (7.4)  [14] | 28.0 (5.5)  [41] | 0.015 | 0.002 | 0.61 |
| **Mean lowest PCR Ct value detected, (SD)**^d^ | 22.0 (4.8)  [79] | 28.8 (7.2)  [14] | 25.1 (4.9)  [41] | <0.001 | 0.001 | 0.037 |
| **All PCR Ct values ≥30, n (%)**^e^ | 6 (7.6)  [79] | 6 (42.9)  [14] | 7 (17.1)  [41] | <0.001 | 0.113 | 0.050 |
| **Mean days that SARS-CoV-2 RNA was detected by PCR, (SD)^f^** | 18.9 (8.5)  [53] | 13.0 (6.6)  [11] | 17.9 (9.7)  [33] | 0.032 | 0.61 | 0.12 |
| **Mean days that SARS-CoV-2 RNA was detected by PCR with Ct value <30, (SD)^g^** | 7.9 (3.6)  [47] | 6.7 (3.9)  [6] | 7.7 (4.6)  [25] | 0.42 | 0.79 | 0.63 |

SD: Standard deviation.

^a^p-values comparing symptomatic vs. asymptomatic.

^b^p-values comparing symptomatic vs. pre-symptomatic.

^c^p-values comparing asymptomatic vs. pre-symptomatic.

^d^Values relative to the nucleocapsid-1 target.

^e^For nucleocapsid-1 and -2 targets.

^f^Time from diagnosis to first of two consecutive swabs with Ct ≥40 on both nucleocapsid-1 and -2 targets.

^g^Time from diagnosis to first of two consecutive swabs with Ct ≥30 on both nucleocapsid-1 and -2 targets.

**Figure-S1.** Epidemiological curves

**A**

Frequency of PCR-confirmed SARS-CoV-2 infection among hospitalized patients at Chris Hani Baragwanath Academic Hospital.

**B**

Weekly PCR-confirmed SARS-CoV-2 infection per 1000 HCWs enrolled in the study.

**Figure-S2.** Distribution of length of PCR positivity according to symptomatology.


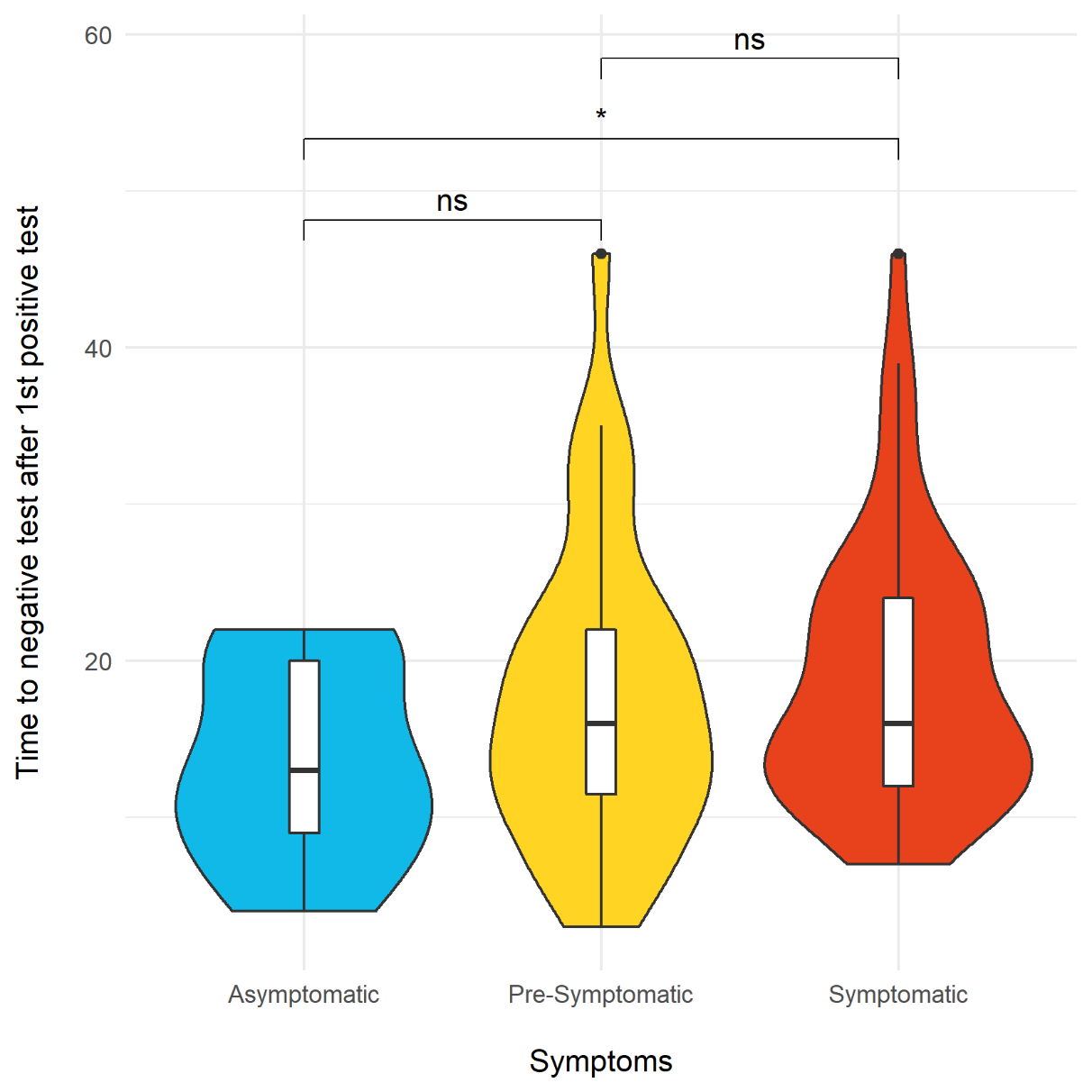


Days from collection of the first PCR-positive swab to the time of the first of two consecutive negative swabs as a proxy for the duration of SARS-CoV-2 infection. Asymptomatic infections were statistically significant shorter compared to symptomatic infections. *p=0·003

References

1. Stadlbauer D, Amanat F, Chromikova V, Jiang K, Strohmeier S, Arunkumar GA, et al. SARS-CoV-2 Seroconversion in Humans: A Detailed Protocol for a Serological Assay, Antigen Production, and Test Setup. Current protocols in microbiology. 2020;57(1):e100.

2. Simani OE, Izu A, Violari A, Cotton MF, van Niekerk N, Adrian PV, et al. Effect of HIV-1 exposure and antiretroviral treatment strategies in HIV-infected children on immunogenicity of vaccines during infancy. Aids. 2014;28(4):531-41.

3. Bullard J, Dust K, Funk D, Strong JE, Alexander D, Garnett L, et al. Predicting infectious SARS-CoV-2 from diagnostic samples. Clin Infect Dis. 2020.

4. La Scola B, Le Bideau M, Andreani J, Hoang VT, Grimaldier C, Colson P, et al. Viral RNA load as determined by cell culture as a management tool for discharge of SARS-CoV-2 patients from infectious disease wards. Eur J Clin Microbiol Infect Dis. 2020;39(6):1059-61.

5. http://www.statssa.gov.za.

6. Lumley SF, Wei J, O'Donnell D, Stoesser NE, Matthews PC, Howarth A, et al. The duration, dynamics and determinants of SARS-CoV-2 antibody responses in individual healthcare workers. Clin Infect Dis. 2021.
